# Affinity maturation of humanized anti-epidermal growth factor receptor antibody using a modified phage-based open sandwich selection method

**DOI:** 10.1038/s41598-018-23796-3

**Published:** 2018-04-03

**Authors:** Hideaki Sanada, Kazuki Kobayashi, Kenji Oyama, Takamitsu Maru, Takeshi Nakanishi, Mitsuo Umetsu, Ryutaro Asano, Izumi Kumagai

**Affiliations:** 1 Department of Biomolecular Engineering, Graduate School of Engineering, Tohoku University, Sendai, 980-8579 Japan; 2 Present Address: Department of Biotechnology and Life Science, Graduate School of Engineering, Tokyo University of Agriculture and Technology, Tokyo, 184-8588 Japan

## Abstract

Affinity maturation is one of the cardinal strategies for improving antibody function using *in vitro* evolutionary methods; one such well-established method is phage display. To minimise gene deletion, we previously developed an open sandwich (OS) method wherein selection was performed using only phage-displaying VH fragments after mixing with soluble VL fragments. The decrease in anti-EGFR antibody 528 affinity through humanization was successfully recovered by selecting VH mutants using this OS method. However, the affinity was not similar to that of parental 528. For further affinity maturation, we aimed to isolate VL mutants that act in synergy with VH mutants. However, the OS method could not be applied for selecting VL fragments because the preparation of soluble VH fragments was hampered by their instability and insolubility. Therefore, we initially designed a modified OS method based on domain-swapping of VH fragments, from added soluble Fv fragments to phage-displaying VL fragments. Using this novel Fv-added OS selection method, we successfully isolated VL mutants, and one of the Fv comprising VH and VL mutants showed affinity almost equivalent to that of parental 528. This method is applicable for engineering other VL fragments for affinity maturation.

## Introduction

Hybridoma^1^ and humanization^2,3^ remain the major practical techniques used for obtaining specific antibodies and for their application as therapeutics, respectively. One of the major methods in humanization is complementarity-determining region (CDR) grafting, in which all six CDRs of the variable heavy domain (VH) and light domain (VL) derived from non-human antibodies, such as mouse and rat antibodies, are grafted on CDRs of appropriate antibody sequences derived from humans^4^. Although humanization, i.e. fabrication of a fully non-natural chimeric protein, often involves a severe reduction in affinity^2,5^, several trial-and-error studies have been reported thus far to improve the affinity of humanized antibodies^6,7^.

*In vitro* evolutionary methods involving various display technologies using phages^8^, yeast^9^, bacteria^10^, and ribosomes^11^ are a powerful tool and have been applied in antibody engineering^12^. In particular, phage display is often used in affinity maturation of antibodies, antibody humanization, and approving the antibody as a clinical reagent^13,14^. Single-chain Fv (scFv) has been widely used in a fragment antibody format for phage display; however, it poses concerns related to gene deletion. To minimise the size of the loaded fragment antibody on the phage for preventing gene deletion, we previously developed the open sandwich (OS) selection method, in which selection was performed using a phage displaying only VH fragments, after mixing with soluble VL fragments^15,16^. This method has resulted in successful antibody engineering, such as isolation of antibodies with specific conversion and affinity maturation^17–19^.

Epidermal growth factor receptor (EGFR) is a transmembrane tyrosine kinase receptor widely expressed in various solid tumours. Because its expression level is correlated with malignancy, metastatic phenotype, and poor prognosis, EGFR is a promising target molecule for cancer immunotherapy^20–22^. In the present study, we focused on anti-EGFR antibody 528 and reported marked anti-tumour activity of bispecific diabody (bsDb) comprising variable regions from mouse 528 (m528) and anti-CD3 antibody OKT3 (Ex3)^23^. After the construction of humanized 528 (h528), we integrated it into several recombinant bispecific antibody formats, such as single-chain diabody and tandem scFv, including their Fc fusion formats, and reported its functionality and usability^24,25^. In our study, we also reported reductions in the affinity of 528 by humanization^26^. Although we successfully increased the affinity of h528 by introducing random mutations into the VH region followed by selection using the OS method, the affinity was not yet equivalent to that of parental 528^19^.

Here, for further affinity maturation, we attempted to isolate h528 VL mutants that could synergistically act with VH mutants previously isolated by us. However, the OS method could not be applied for selecting VL fragments because the preparation of soluble VH fragments was hampered by their instability and insolubility. Thus, we designed a modified OS method based on domain-swapping of VH fragments, from added soluble Fv fragments to phage-displaying VL fragments. Using this novel Fv-added OS method, we successfully isolated h528 VL mutants with high affinity. This method may also be useful for engineering antibody VL fragments and integrating isolated high-affinity VL mutants into engineered antibodies previously constructed by us based on h528 Fv^19,27,28^ for increasing their affinity and tumour-inhibitory effects.

## Results

### Designing the Fv-added OS selection method for VL affinity maturation

**Figure 1:**
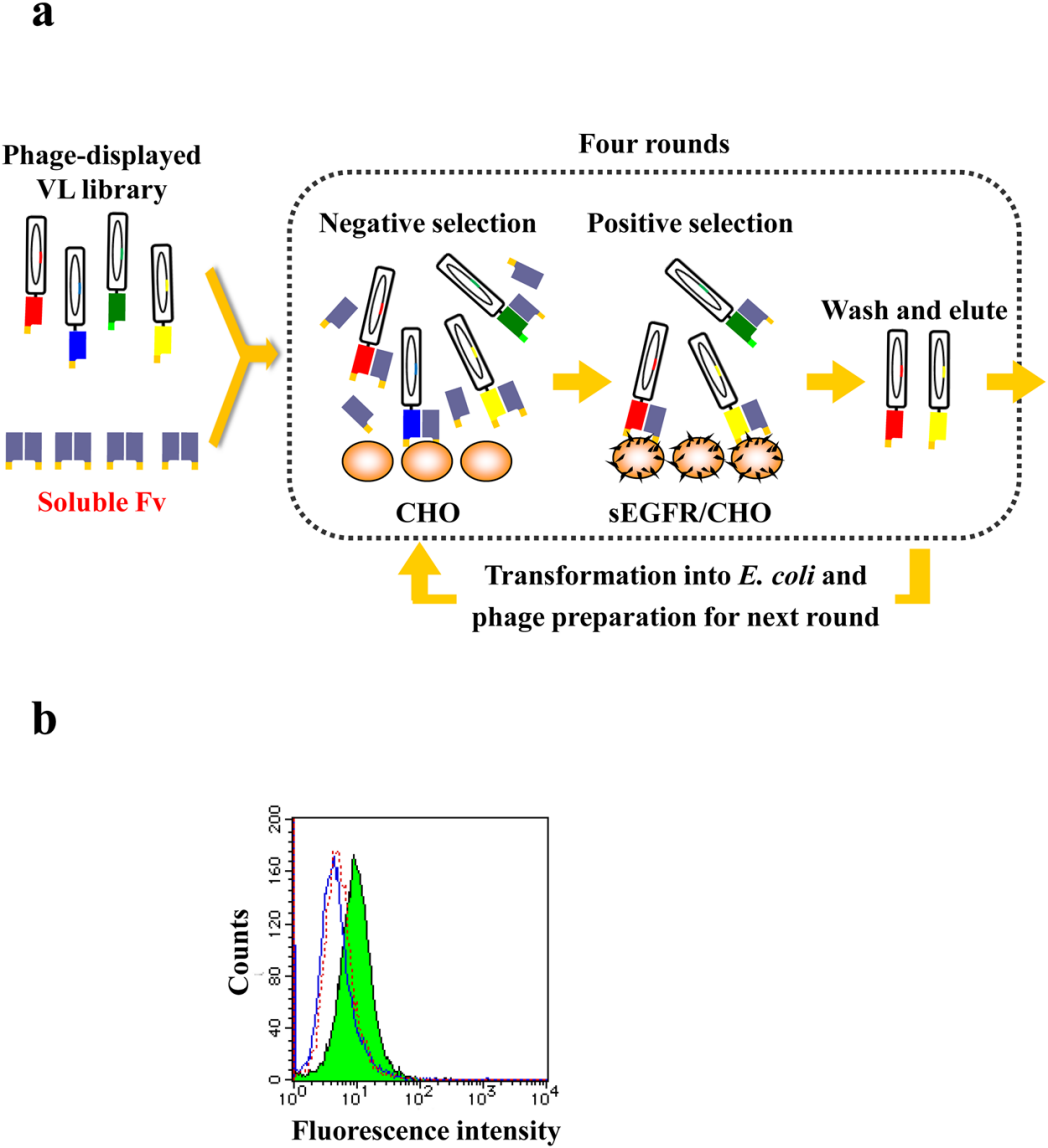
(**a**) Schematic illustration of the Fv-added OS selection method for VL selection. Soluble 2HH11 Fv were added into the phage-displaying VL library, and selection was performed against normal CHO cells as the negative selection. Unbound phages were used for a subsequent positive selection against EGFR-overexpressed CHO cells. After four rounds of selection, clones were isolated and sequenced. (**b**) Confirmation of domain swapping of h528 Fv using flow cytometry. h528 Fv without peptide tag (solid line) and h528 VL (dotted line) with c-Myc tag were incubated with A431 cells and stained with FITC-labelled anti-c-Myc antibody. Both antibody fragments were mixed and immediately incubated with A431 cells followed by staining with FITC-labelled anti-c-Myc antibody (filled area).

For affinity maturation of h528 VL, we designed a novel Fv-added OS selection method. For h528 VH maturation, we used a previously described VL-added OS selection method^19^. In short, to prevent gene deletion and to minimise the size of the loaded protein on the phage, we used an h528 VH phage-displaying domain mutant library. After the addition of soluble VL fragments prepared using *E. coli*, selection was performed, and high-affinity h528 VH mutants were successfully isolated^19^. For VL selection, however, this OS method could not be applied because the preparation of soluble VH fragments was not possible owing to their instability. Thus, we designed a modified OS selection method based on domain swapping, using soluble Fv fragments instead of VH fragments (Fig. 1a). To confirm h528 VH domain-swapping from soluble h528 Fv to h528 VL-display on the phage, flow cytometric analysis was performed against EGFR-positive A431 cells using soluble h528 Fv fragment without tag and h528 VL fragment with a c-Myc-tag. The VL fragment showed results comparable to those of undetectable Fv fragment, indicating that the VL fragment *per se* did not exhibit any binding activity (Fig. 1b). We also confirmed the undetectable binding of the VH fragment (Supplementary Fig. 1). In contrast, a mixture of these two fragments showed obvious binding activity, although analysis was performed immediately after mixing. These results indicate that domain swapping of h528 VH occurs quickly, and that this Fv-added OS selection method can be applied for the affinity maturation of VH fragments.

### Construction of CDR library and selection of high-affinity mutants

**Figure 2:**
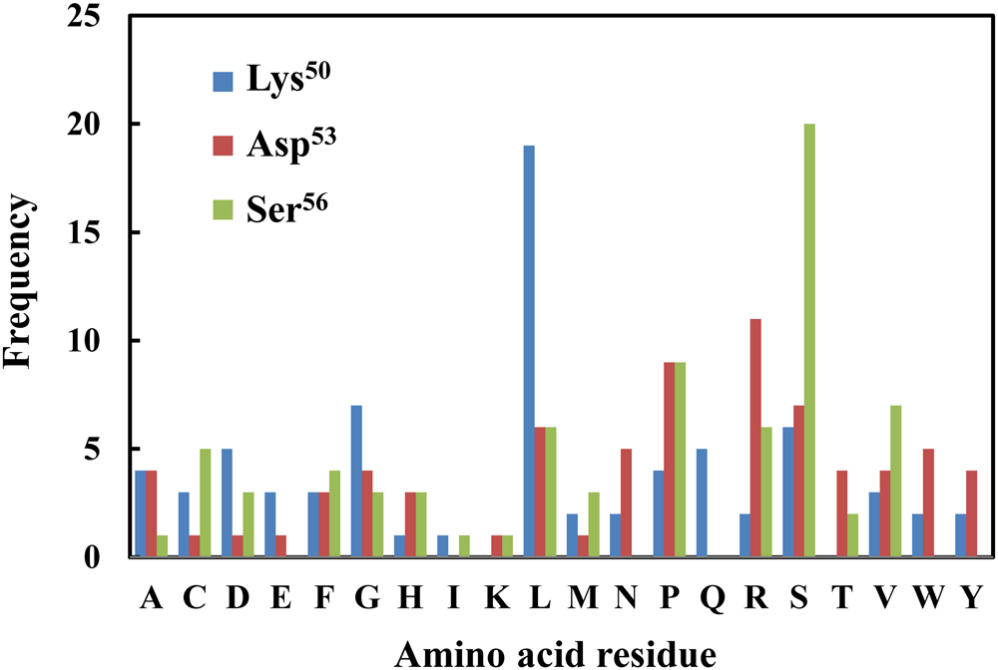
Output amino acid frequency at an Fv concentration of 10 nM.

To improve the affinity of h528 VL, we constructed a CDR-focused library and applied the Fv-added OS selection method. Based on the crystal structure of h528 Fv^26^, we constructed a VL library with random mutations at Lys^50^, Asp^53^, and Ser^56^ in CDR-L2 (Table 1). These sites were located on the solvent-accessible surface, and we restricted the mutation to only three residues to ensure theoretical diversity. Mutations were introduced by the means of an overlap PCR method using oligonucleotides containing the NNK codon at the mutation sites, and prior to selection, DNA sequencing was performed to confirm that the clones from the library were not biased. The 1 × 10^13^–10^14^ phage particles were mixed with soluble h528 Fv 2HH11, which comprised previously isolated VH 2HH11, with a *K*_A_ of approximately 5 × 10^8^ M^−1^ for EGFR^19^. Next, negative selection using normal Chinese hamster ovary (CHO) cells, followed by positive selection using EGFR-expressing CHO cells was performed. We performed selection experiments independently four times at different concentrations of 0.1 nM, 10 nM, 1 μM, and 100 μM of soluble Fv. After four rounds of selection, we analysed the amino acid sequences of the clones randomly selected from each condition, and obtained four (2L1–2L4) and one (2L5) enriched clones from 10 nM and 100 μM soluble Fv concentrations, respectively (Table 1). No enriched clones were observed at concentrations of 0.1 nM and 1 μM Fv. The output amino acid frequency with 10 nM Fv is summarised in Fig. 2. High frequencies of Leu and Arg were observed at Lys^50^ and Asp^53^, respectively, and the wild-type (WT) residue was observed frequently at Ser^56^. Thus, we also selected an unenriched clone 2L6, comprising only residues with a high frequency. It is notable that no WT clones were found in this condition. These results showed that enriched h528 VL mutants were successfully isolated using the novel Fv-added OS selection method.

**Table 1:** Sequence of selected clones at the mutated sites.

Clone	Frequency	Mutated site		
		50^a^	53	56
WT	0/74^b^	K	D	S
2L1	2/74^b^	L	R	L
2L2	2/74^b^	L	A	R
2L3	2/74^b^	Q	S	S
2L4	2/74^b^	S	S	H
2L5	2/72^c^	V	W	R
2L6	1/74^b^	L	R	S

^a^The numbering of residues was based on the study of Kabat *et al*.^42^.

Frequency at soluble Fv concentrations of ^b^10 nM and ^c^100 μM.

### Preparation and evaluation of Fv with isolated VL mutants

**Figure 3:**
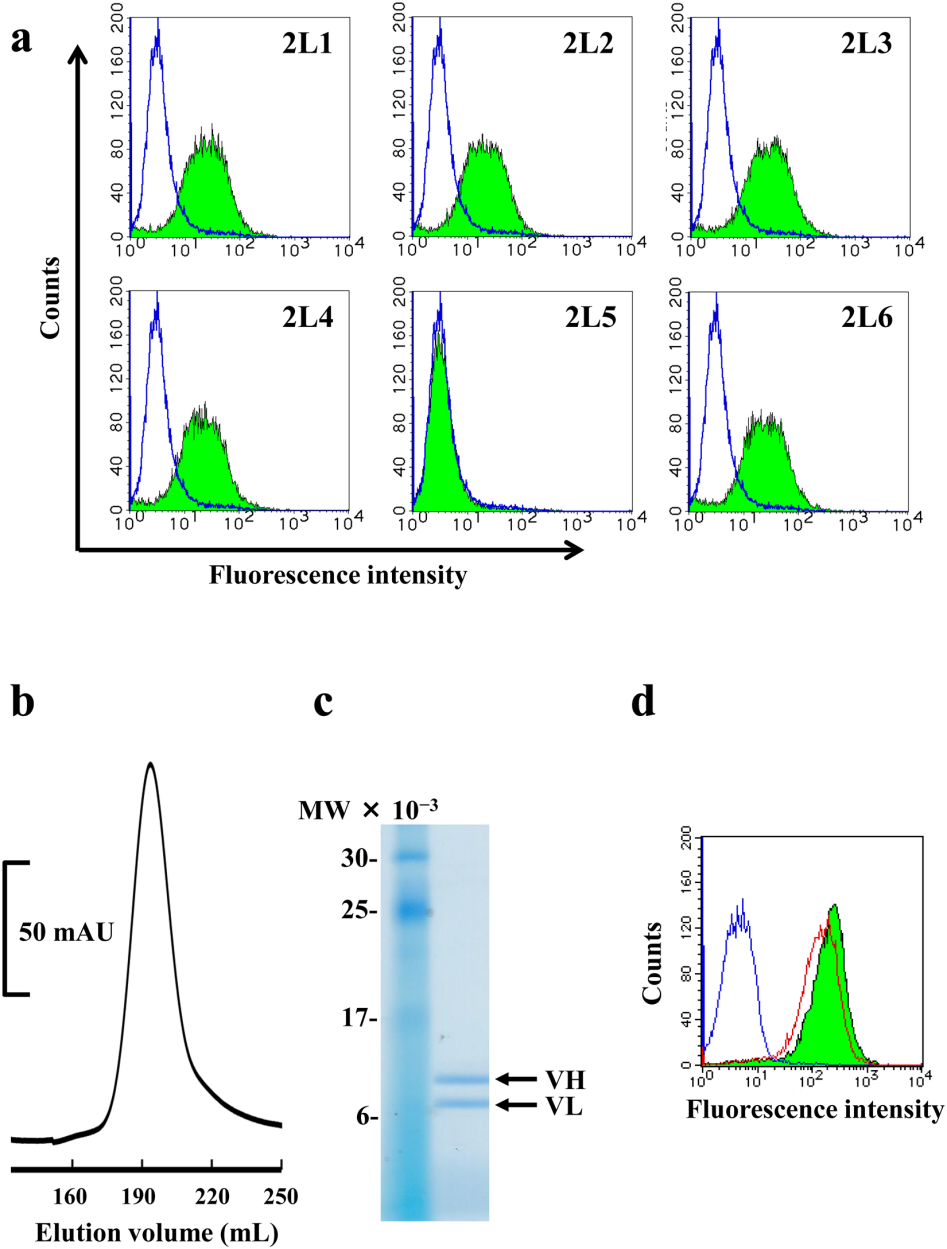
(**a**) Binding property of Fv with selected VL mutants. A431 cells were incubated with PBS as the negative control (open area) or with each intracellular soluble fraction of Fv with VL mutations (filled area); incubation was followed by staining with FITC-labelled anti-c-Myc antibody. (**b**) Gel filtration of Fv (2HH11 + 2L1) purified through immobilised metal affinity chromatography. mAU, milli-absorbance unit. (**c**) SDS-PAGE analysis of the eluted fraction under reducing conditions. The full-length image is presented in Supplementary Fig. 3. The calculated molecular masses for VH and VL are 16.2 kDa and 13.4 kDa, respectively. (**d**) Binding property of purified Fv with selected VL mutants. A431 cells were incubated with PBS as the negative control (blue solid line), with parental m528 Fv as the positive control (red solid line), or with purified Fv (2HH11 + 2L1) (filled area); incubation was followed by staining with FITC-labelled anti-c-Myc antibody.

To evaluate the binding property using flow cytometric analysis, Fv with isolated VL mutants were prepared through the bacterial expression system. Co-expression vectors containing VH 2HH11 and each VL mutant were constructed, and the transformant *E. coli* cells were cultured separately. Results of flow cytometric analysis using intracellular fractions showed that binding activities to EGFR-positive cell line were observed in all selected clones, except for 2L5 (Fig. 3a). For further evaluation, we selected two clones, 2L1 and 2L6, and purified them by immobilised metal affinity chromatography followed by gel filtration because these clones contained multiple high-frequency residues. In both the clones, monodisperse peaks were observed in gel filtration and SDS-PAGE of the eluted fraction, thereby demonstrating the high purity and stoichiometric association. Results for Fv (2HH11 + 2L1) are shown in Fig. 3b,c as representative examples. We also confirmed the comparable binding property of the mutants with parental m528 Fv by flow cytometric analysis using the purified samples. Results for Fv (2HH11 + 2L1) are shown in Fig. 3d as a representative example. These results showed that h528 Fv reconstituted with VL mutants selected by the novel method based on VH domain swapping can be adopted as the uniform format for Fv preparation.

### Thermodynamic analysis of the interaction between sEGFR and h528 Fv mutants

**Figure 4:**
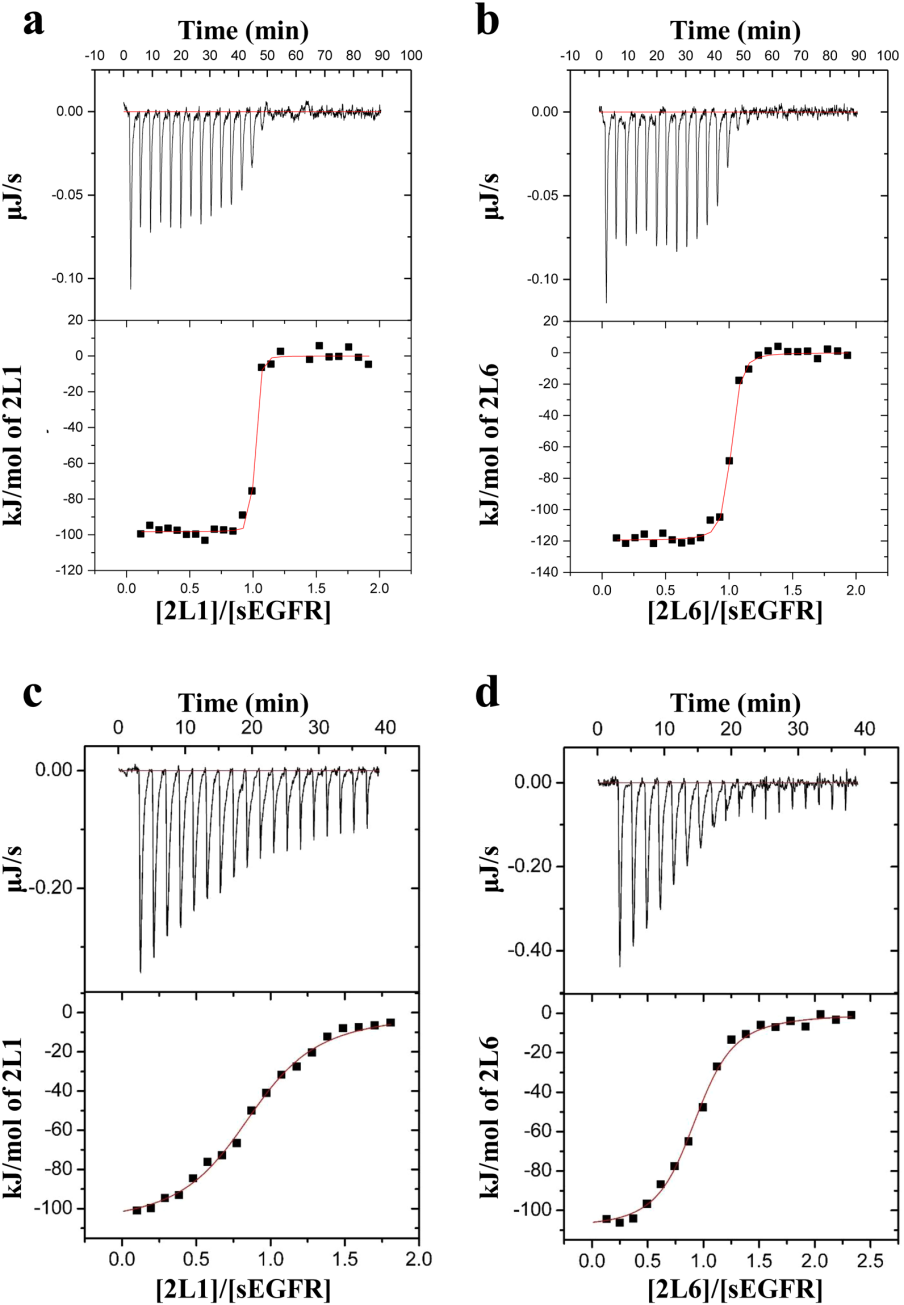
Titration calorimetry of the interaction between h528 Fv mutants and sEGFR. Calorimetric titration of 2L1 (**a**) and 2L6 (**b**) at 25 °C is shown. Displacement calorimetric titrations of 2L1 (**c**) and 2L6 (**d**) with EGF at 25 °C are shown as representative graphs.

To investigate the interaction between sEGFR and the selected Fv mutants, we performed kinetic analyses. First, we determined the binding kinetics using immobilised sEGFR and surface plasmon resonance spectroscopy (Supplementary Fig. 2 and Table 1). Rise in binding affinity was observed in both the mutants; however, we assumed that the reliability of these values was low because the dissociation phases were very slow, which would affect accurate fitting. Next, we performed thermodynamic analysis by isothermal titration calorimetry (ITC) (Fig. 4a,b). Thermodynamic parameters at 25 °C, calculated from titration curves, are summarised in Table 2. Substantial increases in binding affinity constants (*K*_A_) were observed in both h528 Fv mutants (Table 2). However, we also assumed that the reliability of these values was low because the titration curves for both mutants were very steep. To accurately evaluate the binding constants, we performed displacement calorimetric titration with EGF as the weak ligand according to a previous report (Fig. 4c,d)^29^. Thermodynamic parameters with comparability were successfully obtained and are summarised in Table 3. Rise in the binding affinity was observed in both the mutants, and 2L6 exhibited an affinity almost equivalent to that of parental m528 Fv. By screening h528 VL mutants, further enhancement of h528 affinity was accomplished, and we expect the resultant h528 Fv mutants to further improve our engineered antibodies based on h528 Fv for cancer therapy^19,27,28^.

**Table 2:** Thermodynamic parameters of the interactions between Fv and sEGFR at 25 °C.

Clone	*K*_A_ (×10^7^ M^−1^)	Δ*G* (kJmol^−1^)	Δ*H* (kJmol^−1^)	*T*Δ*S* (kJmol^−1^)
m528 Fv^a^	81.7	−50.9	−80.0	−29.1
2HH11 + WT	54.9	−49.9	−87.8	−37.9
2HH11 + 2L1	410.0	−54.8	−99.9	−45.1
2HH11 + 2L6	105.2	−51.5	−108.7	−57.2

*K*_A_, binding constant; Δ*G*, Δ*H*, and Δ*S*, changes in Gibbs energy, binding enthalpy, and entropy, respectively.

^a^Data from our previous work are shown^26^.

**Table 3:** Thermodynamic parameters of the interactions between Fv and sEGFR at 25 °C from the displacement titration method.

Clone	*K*_A_ (×10^7^ M^−1^)	Δ*G* (kJmol^−1^)	Δ*H* (kJmol^−1^)	*T*Δ*S* (kJmol^−1^)
EGF	1.5	−41.0	39.6	80.6
m528 Fv	86.5	−51.0	−88.2	−37.2
2HH11 + WT	48.9	−49.6	−80.0	−30.4
2HH11 + 2L1	56.6	−50.0	−70.0	−20.0
2HH11 + 2L6	86.0	−51.0	−70.5	−19.5

## Discussion

Phage display, a well-established and sophisticated method^30–32^, is a type of affinity maturation method for improving antibody functions using *in vitro* evolutionary methods. Moreover, we previously tried to improve the decrease in affinity of anti-EGFR antibody 528 by humanization using a phage display-based OS method^19,26^. The affinity was successfully recovered by selecting VH mutants, and an increase in cancer growth inhibition was observed by integrating the mutants into bsDb format. However, the affinity was not equivalent to that observed in parental mouse 528^19^.

In this study, for further affinity maturation, we selected VL mutants that act in synergy with 2HH11 VH, one of the promising h528 VH mutants^19^. However, application of the OS method was difficult for VL selection owing to the instability and insolubility of soluble VH fragments. In fact, the recombinant expression of h528 VH fragment using *E. coli* was observed primarily in the insoluble fraction (unpublished data). Therefore, we designed a modified OS method based on domain swapping between VH and VL (Fig. 1a). This type of swapping has been reported previously^33,34^, and in this case, immediate domain swapping between the VL fragment in h528 Fv and soluble VL was observed by flow cytometry (Fig. 1b). Thus, we applied the Fv-added OS method for the selection of VL mutants.

In affinity maturation of antibodies, library design is one of the key points, and examining the 3D structure of the antibody–antigen complex provides critical information. However, we have succeeded in performing structural analysis only for h528 Fv. Thus, based on the theory that CDRs play an important role in antibody affinity and specificity^35,36^, we decided to introduce CDR residues in the 528 VL fragment. The theoretical diversity of a library increases exponentially with the number of randomised residues. In fact, we previously enhanced the affinity of antibodies using small phage-display libraries successfully^19,37^. Therefore, we restricted our analysis to three residues located on CDR-L2, which has a large solvent surface area. The sequence analyses of approximately 100 randomly selected clones from the library before selection showed that almost all amino acids appeared at each mutation site (data not shown).

We selected the mutants under four conditions with different concentrations of added Fv at 0.1 nM, 10 nM, 1 μM, and 100 μM, and obtained sequence-enriched clones primarily at the concentration of 10 nM (Table 1). Only one enriched clone, 2L5, was isolated at the concentration of 100 μM. However, this clone did not exhibit affinity to the EGFR-positive cell line (Fig. 3a), indicating that 2L5 may merely present high display efficiency on the phage and high growth efficiency in *E. coli*. Considering the input titre of the phage and possible display efficiency, the concentration of 10 nM reflected an almost equal ratio of phage-displaying VL to added Fv. Although the number of phage-displaying functional Fv was too low to be selected at low Fv concentrations, addition of excess Fv may induce a competitive environment. Moreover, the actual reason of enrichment of the functional clones, which was not observed under other conditions, is still unknown. Thus, these results show that the concentration of added Fv would affect clone selection in our method.

We also selected one unenriched clone, 2L6, because it constituted only residues with a high frequency at each mutation site (Fig. 2; Table 1). Flow cytometric analysis using intracellular fractions of *E. coli* harbouring co-expression vectors containing 2HH11 VH and each selected VL mutant revealed the binding activities related to the EGFR-positive cell line, except for 2L5 (Fig. 3a). For further evaluation, we selected 2L6 and 2L1, which also primarily comprised residues with a high frequency but did not harbour WT sequences (Fig. 2; Table 1). Thermodynamic analyses using successful highly purified samples (Fig. 3b,c) showed substantial increases in affinity, particularly for 2L1 (Table 2). However, we assumed that these values were unreliable because both the inflection points in titration curves were very sharp (Fig. 4a,b). Thus, we performed a displacement calorimetric titration with EGF as the weak ligand according to a previous report^29^, and successfully obtained thermodynamic parameters from well-fitting titration curves (Fig. 4c,d; Table 3). Displacement titration is one of the potent methods to evaluate relatively strong interactions close to the detection limit of ITC, and we could confirm that the affinity of 2L6 was almost equivalent to that of parental 528 Fv.

Here, we established a novel Fv-added OS method for affinity maturation of VL fragments and successfully isolated high-affinity VL mutants that acted synergistically with previously prepared VH mutants. Humanization remains one of the practical methods to apply murine antibodies for therapeutic purpose. In fact, almost half of the approved antibodies are developed through humanization^38^. However, it often involves a severe reduction in affinity^2,5^ and requires affinity recovery^6,7^. We propose that the method developed by us may be useful for engineering VL fragments for affinity maturation. Furthermore, we previously confirmed cytotoxic enhancement of Ex3 bsDb for cancer growth inhibition by integrating high-affinity VH mutants^19^, and have reported several recombinant cancer therapeutic antibodies based on h528^27,28,39,40^. These cytotoxic enhancements can also be made by integrating the isolated VL mutants into VH mutants and may result in the development of more desirable molecules.

## Methods

### Construction and preparation of the phage-displaying VL library

The CDR-L2 library was prepared in accordance with the methods for construction of CDR-H2 library reported previously^19^. Based on the results of the crystal structure of h528 Fv^26^, we selected three residues located on the solvent-accessible surface of h528 CDR-L2. DNA fragments encoding h528 VL with three randomised residues were generated by overlap extension PCR, and subsequently cloned into the phagemid vector pTZ^41^. The phage-displaying VL library was prepared using the helper phage M13KO7 as described previously^37^. Phages were precipitated using 20% polyethylene glycol 6000 with a 2.5 M NaCl solution, centrifuged, and suspended in 1 mL phosphate-buffered saline (PBS) with 0.1% NaN_3_ per 20 mL of culture.

### Selection of high-affinity VL mutants

We have previously reported successful isolation of the high-affinity h528 VH mutant 2HH11^19^. For affinity maturation of h528 VL, soluble h528 Fv with 2HH11 mutation (h528 Fv 2HH11) was expressed and produced in *E. coli* according to a previous report^19^. Next, a 1-mL aliquot of phage-displaying VL library was mixed with 100 μL of 0.1 nM, 10 nM, 1 μM, or 100 μM soluble h528 Fv 2HH11. After incubation for 1 h at room temperature, each mixture was added to EGFR-negative CHO cells (1 × 10^6^), and incubated for 1 h for negative selection. The reaction mixtures were centrifuged and the supernatant was added to EGFR-overexpressing CHO cells (1 × 10^6^), and incubated for 1 h for positive selection. Next, the cells were washed five times with 1 mL PBS containing 0.02% Tween 20. The bound phages were eluted using 500 μL of 0.1 M glycine-HCl (pH 2.0), neutralised to pH 8.0 with 2 M Tris base. Subsequently, the eluted phages were amplified in *E. coli* JM109 as described previously^37^. After four rounds of selection, the nucleotide sequences of the VL gene from randomly isolated phage clones from different Fv conditions were determined by DNA sequencing.

### Preparation of Fv with isolated VL mutants

Selected VL genes were cloned into a T7 promoter-based pRA expression vector, with a VH 2HH11 gene to construct co-expression vectors for Fv^19^. Preparation and purification of h528 Fv mutants were performed as described previously^19,26^. *E. coli* BL21 (DE3) cells transformed with each co-expression vector for Fv with selected VL mutants were cultured at 28 °C in 2 × YT broth. When the optical density at 600 nm was 0.8, 1 mM isopropyl-1-thio-b-D-thiogalactopyranoside (IPTG) was added to the culture to induce protein production, and the cells were further incubated overnight. The culture supernatant was concentrated by salting out with ammonium sulphate at 80% saturation. The purification was performed by immobilised metal affinity chromatography using a Ni sepharose 6 fast flow column (GE Healthcare Bio-Science, Piscataway, NJ, USA), and gel filtration chromatography using a HiLoad 26/600 Superdex 200 pg column (GE Healthcare) for further purification.

### Flow cytometric analysis

After culturing *E. coli* transformants with co-expression vectors for h528 Fv mutants, cells were separated by centrifugation (6700 × *g* for 20 min). Cell pellets from a 500-μL aliquot of the culture were resuspended in 200 μL PBS and centrifuged after ultrasonication at 150 W for 15 min. A431 human epidermoid carcinoma cells (1 × 10^6^) were incubated with 50 μL of the cell lysate for 30 min on ice. The cells were washed with PBS containing 0.1% NaN_3_, and stained with an anti-c-Myc-tag antibody conjugated with fluorescein isothiocyanate (FITC; 9E10, Santa Cruz Biotechnology, Santa Cruz, CA, USA) for 30 min on ice. The stained cells were analysed using a flow cytometer (FACSCalibur, Becton Dickinson, San Jose, CA, USA).

### ITC

Thermodynamic analyses for the interaction between recombinant Fv and soluble EGFR (sEGFR) were performed by microtitration calorimetry using ITC200 (GE healthcare) as reported previously^19^. All solutions were dialysed against PBS. The solution of sEGFR was placed in a calorimeter cell and titrated against each Fv.

Competitive ITC was performed according to a previous report^29^. sEGFR was first saturated with a weaker binding ligand, EGF, to measure binding parameters directly, and then the ligand was displaced by titration with each Fv. The data were analysed and fitted by the competitive binding model of the MicroCal ORIGIN 7.0 software package.

## Electronic supplementary material

Supplementary information
