# Supplementary material for: Affinity maturation of humanized anti-epidermal growth factor receptor antibody using a modified phage-based open sandwich selection method
Source: Sci Rep. 2018 Apr 3;8:5414. doi: 10.1038/s41598-018-23796-3 (PMC5882652; doi:10.1038/s41598-018-23796-3)
Supplement: Supplementary file 1 — Supplementary information [file 41598_2018_23796_MOESM1_ESM.pdf]

Supplementary information

Affinity maturation of humanized anti-epidermal growth factor receptor antibody using a modified phage-based open sandwich selection method

Hideaki Sanada[1], Kazuki Kobayashi[1], Kenji Oyama[1], Takamitsu Maru[1], Takeshi Nakanishi[1], Mitsuo Umetsu[1], Ryutaro Asano[1,2], and Izumi Kumagai[1]

[1]Department of Biomolecular Engineering, Graduate School of Engineering, Tohoku University, Sendai 980-8579, Japan
[2]Present address: Department of Biotechnology and Life Science, Graduate School of Engineering, Tokyo University of Agriculture and Technology, Tokyo 184-8588, Japan

Supplementary Figure 1

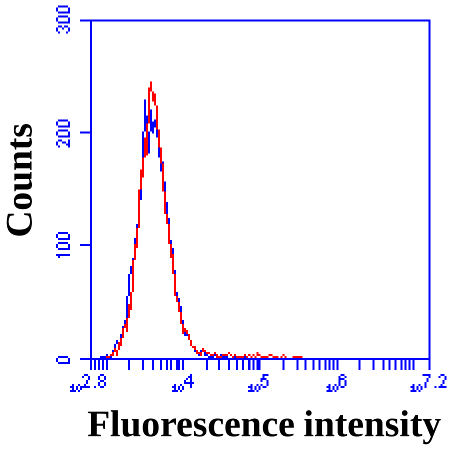

Binding property of the soluble h528 VH fragment. EGFR-positive TFK-1 cells were incubated with PBS as the negative control (blue solid line) or with soluble VH fragment (red solid line); incubation was followed by staining with FITC-labelled anti-c-Myc antibody. The sample was kindly provided by R&D Department of ProteinExpress Co., Ltd. (Chiba 260-0856, Japan)

Supplementary Figure 2

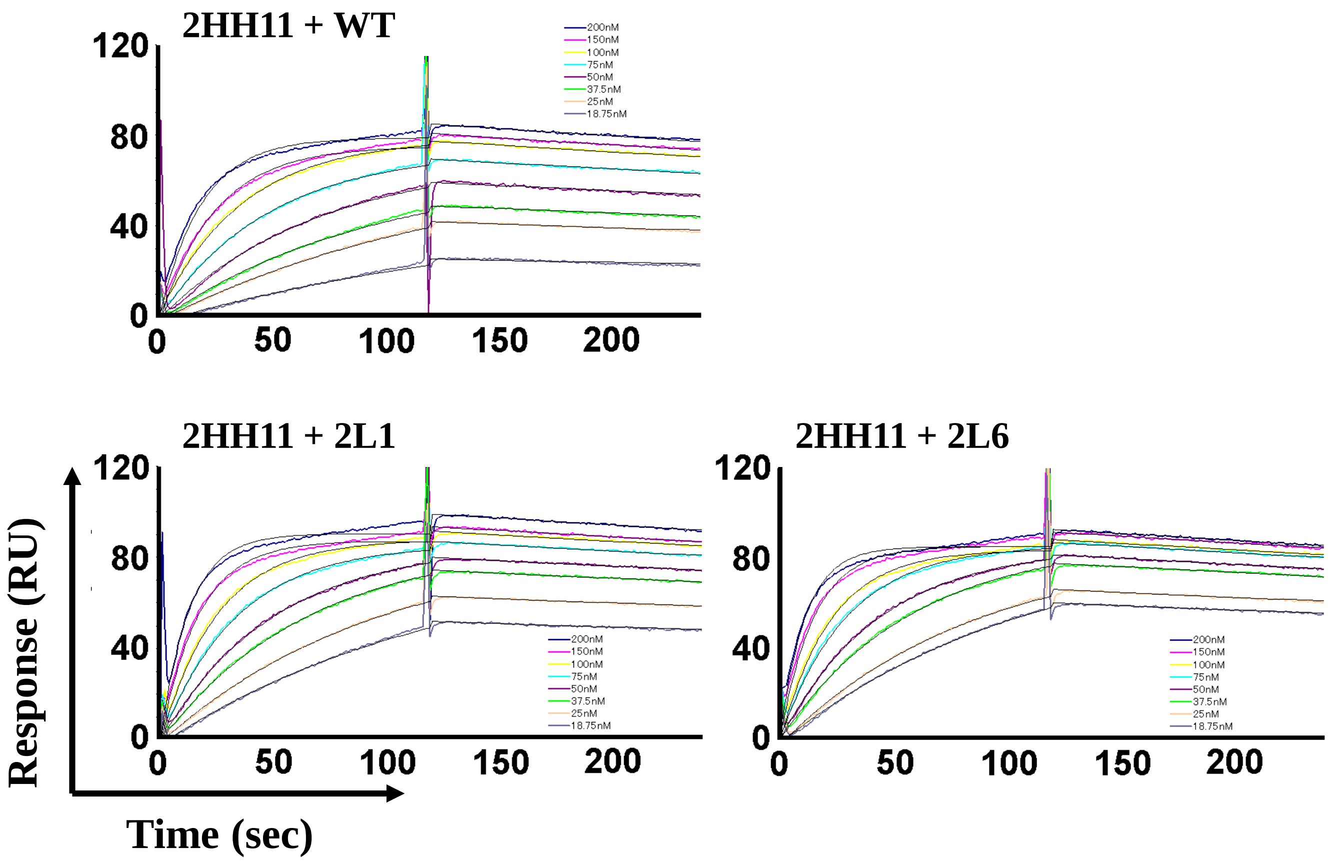

Surface plasmon resonance sensorgrams for h528 Fv mutants. Soluble EGFR was immobilised on a CM5 sensor chip and various concentrations of Fvs were then allowed to flow over the bound EGFR. The data were referenced by subtracting the response of a blocked blank cell.

Supplementary Table 1

Binding parameters. Kinetic parameters were calculated by means of a global fitting analysis with the assumption of a 1:1 Langmuir binding model.

	k_{on} ($\times 10^5$ $M^{-1}s^{-1}$)	k_{off} ($\times 10^{-4}$ s^{-1})	K_A ($\times 10^8$ M^{-1})
**2HH11 + WT**	2.7	8.1	3.3
**2HH11 + 2L1**	3.9	6.0	6.5
**2HH11 + 2L6**	4.5	6.6	6.8

Supplementary Figure 3

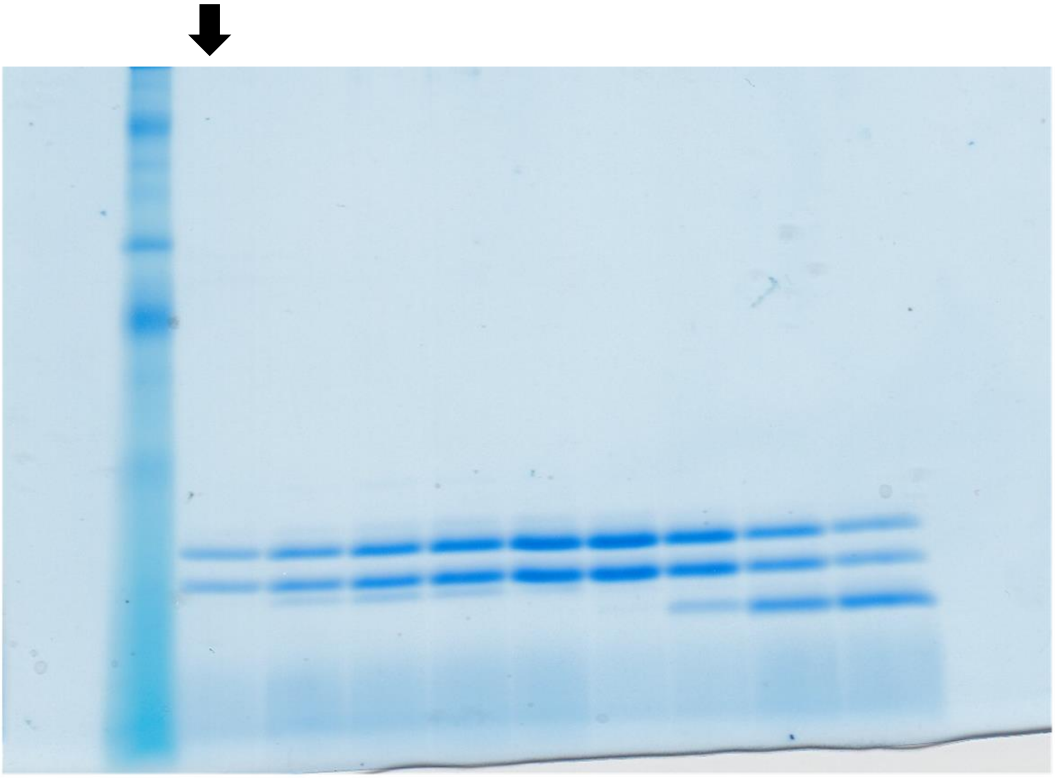

Full SDS-PAGE image of Fig. 3**c.**

The lane indicated by a black arrow represents the eluted fraction shown in Fig. 3C; this sample was used for thermodynamic analysis.
